# Exploring the experiences of female undergraduate nursing students in providing home healthcare to older adults

**DOI:** 10.1080/17482631.2023.2291838

**Published:** 2023-12-06

**Authors:** Wafa Hamad Almegewly, Savvato Karavasileiadou, Taghreed Samel Alotaibi

**Affiliations:** Department of Community Health Nursing, College of Nursing, Princess Nourah bint Abdulrahman University, Riyadh, Saudi Arabia

**Keywords:** Home healthcare, nursing students, experience, older adults, culture

## Abstract

**Background:**

In light of the growing ageing population in Saudi Arabia and Saudi nurses’ hesitation to work with the older population, it is important to understand the experiences of nursing students on their clinical placement in home healthcare.

**Methods:**

This study is a descriptive qualitative study based on five focus groups of 3–5 students each. Data were collected from female senior baccalaureate nursing students at a female governmental university in Saudi Arabia, and during their placement in home healthcare. Data were analysed using thematic analysis.

**Results:**

Five main themes emerged : struggling with their own family; struggling with cultural and social beliefs; struggling during clinical training; struggling with domestic caregivers; and reflections for the future were considered important impressions in clinical experience.

**Conclusion:**

The culture of Saudi Arabian society is an important factor in shaping the training experience of female nursing students either positively or negatively, specifically in the context of home healthcare nursing for older patients. The results of this study show how important it is for students to be prepared for the clinical environment, the nature of the work, and the role of the home healthcare nurse in order to improve their clinical learning.

## Introduction

Home healthcare was established in Saudi Arabia in 1980. Since its launch, home healthcare in Saudi Arabia, has expanded to include a wide range of specialities. These currently include family medicine doctors, registered nurses with home healthcare expertise, dietitians, physiotherapists, and respiratory therapists (Research & King Faisal Specialist Hospital, 2021).; The majority of home healthcare patients are older people and the proportion of older people in the population is set to grow (United Nations, 2019).

Although Saudi Arabia currently has a young population, with only 4% older than 60 years of age according to one estimate, a very high proportion (52.7%) of those over 60 have disabilities and a very high proportion (78.5%) have long term care needs (General Authority for Statistics, 2019). Another estimate, by the UN, measures the proportion of those over 60 years of age in Saudi Arabia in 2017 at 5.6% and predicts it to grow to 22.9% by 2050 (Alghamdi et al., 2020). The government forecasts this will cause strain on hospital capacity that is already stretched by high long-term bed occupancy rates (Frank, 2019). As such, one of the objectives of Saudi Arabia’s national development plan, Vision 2030, is reducing acute long-term bed occupancy by discharging an increasing number of older people and people with long-term conditions into the home healthcare service. To achieve this increased recruitment of qualified nurses into the home healthcare sector is sought. However, one of the obstacles to the success of this policy is the low number of nurses who prefer to work in and be trained in home healthcare services (Alghamdi et al., 2020; Alluhidan et al., 2020).

Statistics show that 78.5% of all older people in Saudi Arabia are dependent on family members in terms of some kind of daily needs help, including mobility and movement (30.9%), self-care (30.6%), medicine taking (24.7%) and eating and drinking (30.9%) (General Authority for Statistics, 2019). The provision of home healthcare therefore can impact the domestic lives of older people with long-term conditions living at home and their families who care for them. The workload of family caregivers is alleviated by domestic workers, although this complicates the use of home healthcare (Alrashed, 2017).

Among the conditions for accessing governmental home healthcare services is that patients and family should identify a primary family caregiver, or alternatively the patient must be able to provide his or her own home care (Research & King Faisal Specialist Hospital, 2021). The initial examination, along with any successive visits by the home healthcare team, must be attended by the family caregiver when the patient needs one (Research & King Faisal Specialist Hospital, 2021). However, many Saudi households depend upon domestic workers who are hired by family members and do not have any special training to do their duties, and who help deliver care to long-term patients (Alrashed, 2017). This runs contrary to the policy that permits only the relatives of the patient to serve as carers during home healthcare visits. Consequently, if a family caregiver is not available, home healthcare service appointments may be postponed even though a domestic worker who provides most of the actual care is present (Research & King Faisal Specialist Hospital, 2021). Although there are many healthcare providers in Saudi Arabia other than governmental (e.g., private, military, royal), everyone has access to free healthcare from the Saudi Ministry of Health.

The Saudi Ministry of Health only provides free home healthcare services for certain diseases. The list of eligible diseases for acceptance includes: patients with cancer, patients who’ve undergone surgeries, Alzheimer’s disease patients, patients who have experienced car accidents or head injuries, psychiatric patients, bedridden patients, wounded people, and patients with bedsores, patients with respiratory conditions, urinary catheterization patients, enteral nutrition patients, patients administered antibiotics intravenously, patients with hypertension, diabetics, patients with cardiovascular diseases (CVDs), patients undergoing physiotherapy and rehabilitation, patients with thrombophilia, patients with hiatal hernia (Ministry of Health, 2021). This can give nursing students training in home healthcare exposure to patients with a wide range of diseases and conditions. However, if we compare it to the hospital experience, home care patients are, on average, relatively unlikely to be in a critical condition. Patients in a critical condition are unlikely to be discharged from hospital. Therefore, for students seeking challenging experience caring for patients with acute, complex needs, hospitals may be seen as the optimal setting for training.

Traditionally, nursing colleges have often relied on hospitals for clinical placements of nursing students and see hospitals as the best practice environments to stimulate and inspire students for their future career (Parvan et al., 2018). This is supported by Aktaş and Karabulut (2016), who claim that students who train in hospitals become more motivated and are more likely to want to specialize in their area of training. This represents a challenge for increasing recruitment numbers into home healthcare.

Understanding the motivations of Saudi nursing students and how they perceive their training experience in home healthcare can assist academic and healthcare planning institutions to devise an effective way to meet increasing demand for home healthcare services. There is little prior research on students’ preferences for training in a home healthcare setting in Saudi Arabia, where the majority of patients are older adults. However, international research suggests that nursing students do not consider older adults to be a desirable patient group to train with (Bisholt et al., 2014; Bjørk et al., 2014). Nursing students in Toronto, Canada, reported positive attitudes towards the care of older adults, but in practice they also reported a low preference for working with older adults (Abudu-Birresborn et al., 2019). There are similar results for training in a home healthcare setting, according to Van Iersel et al. (2018), 76% of nursing students in Amsterdam, the Netherlands, wanted to work in the hospital setting, most often in acute care, emergency, and paediatric departments, whereas community care was preferred by just 8% of students. Home healthcare was perceived as a place for older patients and those with long-term care needs to receive routine care (Bjørk et al., 2014). Students felt home healthcare patients do not need nurses with advanced technical nursing skills (Bjørk et al., 2014). US nurses consider home healthcare a location for training bedside manners and interpersonal skills such as personal communication and relationship-building abilities, particularly when involving older patients (Landers et al., 2016). This may not motivate new graduates since many students want to practice hands-on technical nursing skills and medical procedures (Landers et al., 2016).

In one of the few studies on Saudi nursing students’ preferences, Alsadaan et al. (2021) confirms that they also prefer to train in a hospital environment. Alsadaan implies in the Saudi context this may be driven by cultural factors rather than the development of skills, as observed in research on other countries. The conservatism of Saudi families around gender segregation expresses itself in their influence over the choices of female nursing students to prefer to work in exclusively female hospital environments. Besides, Almegewly et al. (2023) conducted a qualitative study to examine students’ perspectives on pursuing a future career in homecare after training in homecare. Students do not prefer to work in homecare, as they describe it as a routine job that doesn’t offer the prospect of developing their skills. For the majority of students, interesting fields include emergency and acute care.

The present study aims to explore the experiences of female undergraduate nursing students in providing home healthcare to older adults to better understand their preferences in working with older adults. If Saudi Arabia continues to pursue its Vision 2030 plan of increasing the use of home healthcare to reduce acute long-term bed occupancy (Saudi Vision 2030, 2019), action needs to be taken to meet the increasing demand for skills in this area. Older people are both the largest demographic group occupying beds in hospital on a long-term basis in Saudi Arabia and have the greatest demand for home health care. Policy makers in collaboration with nursing schools need to understand how to make older people in home healthcare an attractive group to train and work with if the Vision 2030 policy of increasing the adoption of home healthcare, and therefore the number of Saudi nurses in home healthcare, is to be a success.

Home healthcare nursing training has been introduced and approved for the first time as a clinical placement on the older adults’ course at Princess Nora bint Abdulrahman University (PNU) in Saudi Arabia. It is a university that admits only female students. Despite the fact that home healthcare has been in operation since 1980, the majority of nursing colleges have yet to acknowledge it as a training location. The older adult course at PNU has organized three four-week rotations over 12 weeks for nursing students in hospital wards for older patients, elderly care homes, and home healthcare services. The present study aims to fill a research gap for understanding the views and experience of nursing students who have trained specifically to work with older people.

There is a lack of relevant studies on the databases Medline, PubMed and CINAHL that indicate a low amount of published research investigating student experiences of home healthcare and providing care for older adults in both Saudi Arabia and internationally and the available research does not cover the full variety of student perspectives. Internationally, several studies have focused on components of the learning environment, such as the influence of preceptorship programmes, students’ career planning, and staff nurse assistance without focus on student opinion (Arkan et al., 2018; Günay & Kılınç, 2018; Van Eeden et al., 2016). Other studies focused on workplace quality and variables influencing student teaching were conducted (Algoso et al., 2016; Ming et al., 2019). However, most of the outcomes do not present students’ perspectives and were not based on data from Saudi students.

Furthermore, in Saudi Arabia, there are two studies based on Saudi nursing students’ focusing on home healthcare and older patients’ care. Yousuf (2009) evaluated students’ attitudes towards home healthcare and their level of interest in choosing it as a future career, finding a low level of interest in the geriatric nursing speciality. In contrast, Alsenany (2009) assessed the attitudes and knowledge of nursing students towards the care of older people. Echoing Abudu-Birresborn et al. (2019) findings on Toronto nurses, Alsenany (2009) found that nursing students have a good attitude towards working with older patients. However, the students sampled in the two studies did not train specifically in home healthcare and their opinions were based on caring for older patients in hospital and primary care centre settings.

## Methodology

Descriptive qualitative approach was used to explore the female undergraduate nursing student’s experiences in home healthcare. In order to maintain credibility while reporting the study, The Consolidated Criteria for Reporting Qualitative Research (COREQ) was used (Tong et al., 2007).

### Participants

Participants of this study consisted of 25 Saudi female senior baccalaureate nursing students, who were enrolled as 4^th^ year students who had undertaken the nursing care of older adult course and trained in older adults’ home healthcare for about 11 weeks, 6 hours per 4 visits as part of the course training plan. The study was conducted at one selected governmental female university in Riyadh, Saudi Arabia.

After gaining ethical approval from the selected college of nursing, an official research participation invitation email was sent by the college students’ affairs unit to all the target students’ emails. The interested nursing students who accepted the invitation, communicated with the research team via email to proceed with the research process. In order to avoid coercion, the research team members had no relationship with the students, and a full research information sheet was given to all the interested students as well as provided with sufficient time 48 hours to consider the information before taking part in the study.

### Data collection

All the focus group interviews were conducted by the first and third authors in Arabic and took place in a small private venue in the college of nursing. Five focus group interviews were conducted, including five students each, and lasted for 45 minutes.

**Figure 1.**
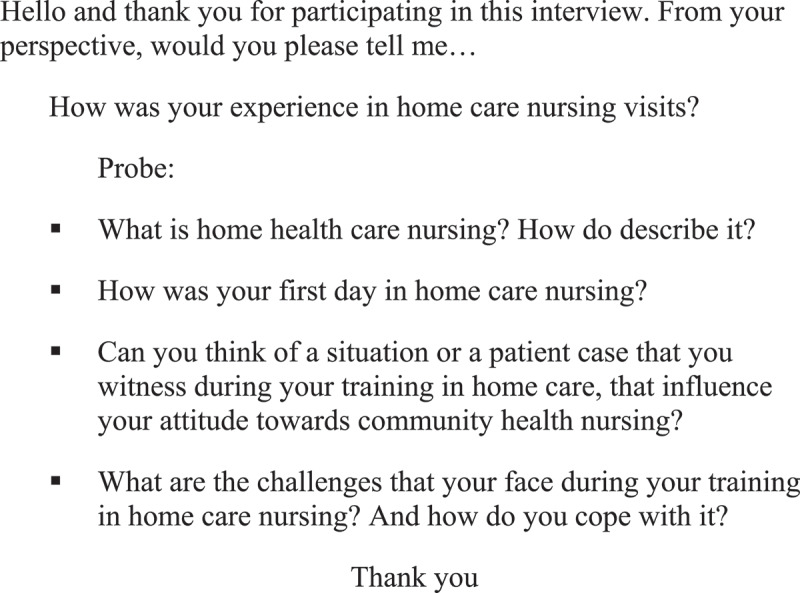
Focus group Interview Guide.

The semi-structured interview guide was retrieved from the relevant literature that focuses on overall nursing students’ experience in home healthcare, the perceived challenges and benefits. The interviews started with an open question: How was your experience in home healthcare nursing visits. The question was followed by probe such as: Can you think of a situation or a patient case that you witness during your training in home healthcare, that influence your attitude towards community health nursing? and can you explain further what you mean. See Figure (1).

All the focus group interviews were audio recorded and verbatim transcribed. Since the third author is bilingual, she has the capacity to translate five focus groups from Arabic to English using verbatim translation and check for accuracy with the first author.

## Ethical consideration

Ethical approval was obtained from Princess Nourah bint Abdulrahman University (ref no:19–0199). Prior interviews, written voluntary consent was obtained by the first author from all the participants, and it was clearly explained that their participation was not part of the course requirement, their marks would not be affected, and they could withdraw from the study at any time. The safeguard procedure was explained to other students and notified that any safety concern will be reported to the students’ affairs.

### Data analysis

The data were analysed by the first and third authors using an inductive, thematic analysis, and semantic approach (Braun & Clarke, 2006). Firstly, we familiarized ourselves with the transcripts by reading and re-reading them. Secondly, an initial list of codes for each transcript was created. The generated codes were compared to each other and categorized into thematic groups. Thirdly, each group, along with the quotes related to the codes, was reviewed to define the themes. Finally, several specific themes and sub-themes that reflect the essence of nursing students’ experiences were developed. See Table I.

**Table I.** Superordinate themes with sub-themes.

*Struggling with their own family*	*Struggling with cultural and social beliefs*	*Struggling during clinical training*	*Struggling with family caregiver*	reflections for future
Hiding their training in home-healthcare Safety issue	Living in fear Saudi culture Getting inside stranger’s (male) house language familiarity	Working with family care givers Language and communication A new area of practice Dealing with older patients	Sense of humanity Violence and abuse Dealing with care givers	Life lessons Setting priorities Thinking of what future hold Thinking of their own parents

## Findings

All the participants were female in their 4^th^ year of their nursing study, had completed their nursing care of older adult course, had trained in older patients’ home healthcare for approximately 9 to 11 weeks, and their age ranged between 20 and 21 years old.

Five main themes were identified: struggling with their own family; struggling with cultural and social beliefs; struggling with clinical training; struggling with domestic caregiver; and reflections for the future.

### Struggling with their own family

Most of the students reported their inability to share their home healthcare training experiences with their families. They preferred to hide it and never mention it to them. Fear of family reaction was the most common reason for hiding their training experience. This fear is mainly associated with safety issues due to the nature of the home healthcare setting that involves getting into different patients’ homes that might be in abandoned remote areas:

My father said that “you are going to strangers’ home … How I can make sure that you are safe there.” (A2)

Some of the students indicated that the fear was amplified by the unavailability of a clinical instructor from the university to accompany them during the home healthcare visits, so they had to go alone. As per the home healthcare regulations, number of students will go to different visits with home healthcare team. Thus, the clinical instructor cannot be with all students once. From a Saudi cultural perspective, other students stated that they come from very “conservative families”, which showed their unwillingness to allow female to enter strangers’ homes, in this case, the “patients” homes”:

“My family is very conservative and has strict rules … I feel like I can’t meet my friends outside. Definitely, they will not allow me to go to patients’ homes that I don’t know”. (C2)

Some students reported that home healthcare is a new concept and society lacks awareness or is yet to comprehend this model of care. Therefore, their parents might not accept the idea of their daughters being trained inside a patient’s residence and away from the known training settings like hospitals or healthcare clinics:

“When my father asked me about the clinical training nature, I told him that I’m doing health education in schools, but he said no (with an anger tone) … “*You’re lying. I know this place very well and your job is not to go to a patient’s home*.” (A2)

### Struggling with cultural and social beliefs

Some cultural issues concerning the position of Saudi female in society and the need to protect them from any potential harm were very evident in some student’s comments. Being trained in home healthcare that involves going to the patients’ homes and not knowing what lies behind closed doors has posed a safety risk, which has caused some students to be fearful:

How come a Saudi girl like me can enter a stranger’s home without knowing what’s inside. (A1)

Moreover, some societal barriers that involve gender segregation and dealing with male caregivers in the patients’ homes but not hospitals, posed another challenge to some students. They were anxious and didn’t provide the required nursing care as they were in direct contact with males:

I went through the same experience. Most of them were men there … I stood aside and I couldn’t do the nursing care. (B2)

On the other hand, some students reported that being Saudis, speaking the Arabic language has positively affected the decision of the patient’s family when it comes to accepting them inside their home. Students commented that patients and their families were more welcoming to them because they can express their needs and communicate with them more effectively than non- Arabic speakers:

The patients were happy because I understood their language because most of the nurses were non-Arabic speakers. (D3)

The patients and their families at home healthcare were very supportive to the Saudi nurses compared to the hospital:

I remember, one lady at the hospital asked me if being a nurse working at the hospital was okay for my parents…! Unlike the older patients here who accepted us and showed their support. (C3)

### Struggling during clinical training

Lack of preparation was evident when a few students reported that they were exposed to incidences where some of the patients harassed them, but they did not know how to react:

I was taking care of a female patient with dementia …when she [the patient] woke up, she started yelling at me, screaming. I got scared. (C3)

“Literally every time we entered a new house, I said in the name of God because I don’t know what is waiting for me inside.” (C3

Some students believed that home healthcare nursing was an unsafe clinical training setting because they could be exposed to potentially dangerous situations, such as violence and harassment from patients or their domestic caregivers:

Emotionally, I will not be able to watch the sad scene daily […] witnessing a close family member neglecting the patient […] and I can’t do or change anything. (A5)

Also, they claimed that they were not safely prepared how to deal with such cases alone, especially within the absence of a clinical instructor during the visit:

The college didn’t prepare us how to deal with such cases like violence or harassment from patients or how to comfort patients. (C2)

Other students shared their concerns about dealing with older patients:

Home healthcare deals mostly with older patients and I’m a very sensitive person … I can’t deal with difficult situations or abuse cases. (A3)

While others described the challenging working nature of home healthcare because of the weather and excessive travel:

I’m suffering from motion sickness. it was difficult for me to stay in the car for a long time during the summer. (A2)

I was feeling tired and nauseated all the time because we were visiting lots of houses. (B4)

### Struggling with family caregiver

Home healthcare nursing enhances the nurturing culture of students those stresses on the importance of taking care of older patients, especially parents, and treating them with dignity and respect. The majority of students were surprised by how people neglected their old and sick mothers or fathers. The state of the neglected parents was simply depressing:

“I witnessed many family neglect incidents there, especially from the caregivers. I felt helpless. I kept quiet; the nurse wrote a report, but I don’t know what happened next. ” (A2)

Many of the older patients received care from home domestic helpers who were privately hired by family members and did not know about their condition, medication, care plan, and wound care. Some students questioned the criteria for selecting the caregiver at home healthcare:

I felt heartbroken when I saw a maid or a caregiver instead of a family member taking care of an old lady or a man. (C2)

I saw an old male patient being taken care of by his driver, and when we asked his wife about his condition. She said, “I don’t know. Ask the driver.” (C5)

They mentioned that some caregivers were not qualified to take care of patients because they were neither family members nor had the proper knowledge and skills in caregiving.

The patient was sleeping on the floor without a bed, and he was being taken care of by a servant who neither spoke Arabic nor English, leading to a serious barrier in communication. His diaper had not been changed for a long time, and he did not take his medication as required. (A1)

On the other hand, returning the favour and taking care of parents was evident in some cases from family members:

The first patient I met had Alzheimer disease, and it was very touching how the son cared for his mother, focusing on every simple detail.

(A3)

### Reflections for the future

The students agreed that training in home healthcare is instrumental in equipping them with some life lessons and enabling them to think and plan for the future. They found that this experience had a positive impact on improving their personalities and enabling them to take care of themselves and think about what matters in life:

“I learned that I’m the one who’s responsible for building my own future; I want to be independent and able to take care of myself when I get older”. (C4)

Witnessing cases of neglect let many students think of what the future holds for them, in terms of who would take care of them when they get old:

“An older patient living in a big house and having no caregiver got me thinking about who would take care of me when I got older and what I could do to help myself when that time comes”. (B3)

Many life lessons were learned from this experience in terms of personality growth, being independent and life priorities, being good with people, and spending more time with their parents, and this was verbalized by many students in the following way:

I felt that this population [older people] was being forgotten. I started to spend more time with my mother. Though she is young, I started to care a lot for her. That is why I can’t re-live this experience again. (B1)

## Discussion

The present study aimed to explore the undergraduate nursing students’ experiences in the older home healthcare environment in Riyadh, Saudi Arabia. Based on the nursing students’ responses, the culture of Saudi society is an important factor in shaping their clinical training experience. Many cultural issues were raised by the students considering the nature of home healthcare work; their family acceptance; their position as Saudi females as well as nurses; and transformational life lessons.

The results showed that nursing students hide their training at older home healthcare away from the family for many cultural reasons related to safety, gender issues, and the nature of clinical training itself. This might be explained by the raised nature of Saudi females who are brought up in a safe environment with a male guardian (Bar, 2017), thus being inside a patient’s home who is considered to be a stranger to her might not be a comfortable experience. The current study showed that nursing students felt uncomfortable delivering care in the presence of family members, especially if they were male. The reason for this is not clear, but it may have something to do with students’ minimal exposure to family interactions during their clinical training (Ford et al., 2016). The society in Saudi Arabia is highly conservative, and families follow strict rules regarding gender mixing outside the family premises. The belief that women are more vulnerable to harassment and may be unable to handle work-related pressures. Therefore, it is difficult for female students to visit, even for work purposes, any house that is not familiar and does not belong to a relative, as this is considered a “strangers” home” (Bar, 2017). Despite that, the current study reported that some patients showed their trust and appreciation after knowing that the nurses were Saudi, sharing the same language and similar beliefs. Given that the majority of the nursing professionals are non-Saudi (Alshammari et al., 2019), the sense of understanding the patient’s needs and the ability to deliver holistic care was highly essential in the nursing student’s experience.

Challenges to the effective delivery of nursing home healthcare are not limited to local cultural trends and beliefs. Worldwide, it has been evident that working with older people in home healthcare, who represent a large percentage of homebound patients, is not an easy task, especially for inexperienced nursing students at the beginning of their professional career. In terms of the clinical nature of older home healthcare training, the current study found that the working environment of home healthcare was challenging because of the weather and excessive visits per day. This finding supports the previous research that found a strong association between home healthcare nurses’ intention to leave their job and environmental factors such as weather and car maintenance (Ellenbecker et al., 2006). Therefore, the high working demands of home healthcare and job benefits or wages should be addressed by policymakers to improve the retention rate of home healthcare nurses (Ellenbecker et al., 2008).

Based on the students’ observations, they reported that the informal caregivers hired by the patient’s family are neither competent nor qualified to take care of older patients. Also, the participants believed that older patients would be at lower risk for healthcare damage if they were taken care of by a family member than an informal caregiver like a housekeeper or a driver. This perception is driven by the Islamic cultural values that emphasize supporting family members in times of need, and these practices have been extended to the next generation. This is evident in a study done by Min and Barrio (2009), who showed that greater proportions of older Mexican American patients compared to non-Latino white ones prefer to depend on close family members because they are more culturally competent than formal ones. On the other hand, different studies suggested that patients should be taken care of by a professional caregiver rather than an informal caregiver to minimize any burdens on the family and, most importantly, to improve patients’ health outcomes.

The current study showed that there were some cases of verbal or physical harassment against some nursing students by patients or their household caregivers. These findings are consistent with other studies which found that home healthcare workers are at high risk for optional hazards that include extreme temperatures, unsanitary conditions, aggressive pets, and physical violence at patients’ homes (Singh et al., 2020). Also, nurses in Saudi Arabia unfortunately experience multiple forms of workplace violence, including verbal, physical, gesture, exclusion, and cyber, coming mainly in the form of patients’ co-workers (Salvador et al., 2021). Similar results were also found in Iran’s context, where healthcare providers were worried about the safety and security of patients when they entered the patients’ homes (Heydari et al., 2016). Home healthcare nurses showed a high level of frustration due to a lack of recognition from managers, though they showed a high level of commitment to their patients’ care (Andresson et al., 2017). Different solutions were suggested to minimize the risk of improper social behaviour against nursing students, like surveillance, support from peers, and clinical preceptors (Park & Jones, 2010; Phoo & Reid, 2022; Quinn et al., 2021). Unfortunately, these measurements have not been applied as some students reflected that they have difficulties in home healthcare because of the absence of clinical instructors and lack of preparation. This supports the importance of clinical presence, which represents the source of security and guides (Turjamaa et al., 2020).

Despite all the cultural and environmental issues, many nursing students still have a positive attitude towards home healthcare. Students were taught many life lessons. By entering older patients’ houses, they occasionally encountered multiple unmet needs and started reflecting on their own current position as daughters towards their older parents and to project themselves into the future as older adults when they will age. These results were consistent with those of Neville (2016), who found that Australian undergraduate nurses reported positive perceptions during working with older adults. These can be explained by the fact that home healthcare provides excellent chances for practicing in a patient-centred and holistic approach to a greater extent than in the hospital environment. Also, students had feelings of compassion and remorse for witnessing some physical neglect and abuse attitudes among older patients, like poor hygiene, safety, and malnutrition. Certainly, older adults’ abuse and neglect is a global phenomenon that home healthcare practitioners and nurses worldwide encounter many times in their professional lives. In Saudi Arabia, however, older patients traditionally maintain their status as leaders of the family and rulers. They receive increased respect and attention from all the members of the family. As per the Islamic religion and cultural customs, older adults are much respected by families and relatives, and neglecting them is considered a sin. This is matched with the results of Almakki et al. (2020), who reported that not meeting the older individuals’ requirements for nutrition, safety, or hygiene is a form of neglect.

One of the limitations of this study that our sample is not absolutely representative of the nursing population in Saudi Arabia, as it is comprised of female students only, coming from a single academic institution.

## Implications

### Nursing education

The feedback from our trainees could provide the basis for a fruitful discussion to identify areas for improvement in the clinical practice and nursing curriculum, to foster a positive attitude and professional behaviour, and improve the quality of home healthcare services in Saudi Arabia. A transformation of the academic curricula towards practical exercise in real clinical settings is required. A structured preparatory course focused on the reality of home healthcare practice, prior to their exposure to the real settings, has become apparent. Progressive involvement of young nursing students from first grade in home healthcare may be a good solution to gradually develop such skills along with endurance to shock experiences and difficulties inherent to home healthcare. Nursing students should be given the chance to debrief after such potentially traumatic events to alleviate any negative feelings such as sadness, create realistic expectations, and improve the student’s skills to successfully manage such events, even alone and away from the safety of the hospital setting.

### Nursing practice

Healthcare policymakers should make home healthcare an attractive working environment for students and develop strategies to increase the retention rate. Religious and cultural values should be an important competence for nurses who provide care for older patients. Both healthcare managers and academic members should work together to facilitate the students’ transition from education to practice. The multiple needs of homebound patients and the special needs of older patients, along with the lack of support and resources, need a systematic, holistic, and problem-oriented approach, the rationale of which can be explained by didactic lectures, but also needs to be reinforced through simulation and role-playing. Preventive measures against harassment and abuse against both patients and students should be considered. To keep patients from getting hurt as much as possible, informal caregivers should be given relevant educational and training programmes in taking care of the older adults.

## Conclusion

This qualitative study provides a valuable insight into the experiences of the female Saudi nursing students during their training in home healthcare. Although the educational potential of the multifaceted care and holistic approach that homecare mandates and rewarding feelings post-interaction with patients and their families were reported, majority of the nursing students had declared anxiety about being alone in a non-clinical setting and identified several difficulties and barriers to their home care practice, especially with regard to their gender and the related traditional perceptions in Saudi Arabia. More importantly, the clinical insecurity in the homecare setting due to the lack of supervision that many nursing students experienced stresses the need for a revision of the academic curriculum towards more preparation and additional onsite involvement of clinical preceptors, in order to ensure both a safe educational environment and the quality of the offered care.
